# The Administration of Levodopa in a Patient With Parkinson’s Disease Using a Novel Maxillofacial Route: A First-in-Human Report

**DOI:** 10.7759/cureus.48011

**Published:** 2023-10-30

**Authors:** Suresh Thirunavukarasu, Balasubramanian Bala Venkata Ramanan, Sathya Krishnan Suresh, Vincent Jayakumar Antonisamy, Devi Varadharaj, Paranjothi Shanmugam, Kavita Verma, Canmany Elumalai, Gladson Selvakumar, Ahila Elumalai, Lydia Prabahar, Hridwik Adiyeri Janardhanan, Anoop UR

**Affiliations:** 1 Neurology, Indira Gandhi Government General Hospital and Post Graduate Institute, Puducherry, IND; 2 Computer Science Engineering, Puducherry Technological University, Puducherry, IND; 3 General Medicine, Indira Gandhi Government General Hospital and Post Graduate Institute, Puducherry, IND; 4 Research and Development, UR Anoop Research Group, Puducherry, IND; 5 Orthodontics and Dentofacial Orthopaedics, UR Anoop Research Group, Puducherry, IND; 6 Conservative Dentistry and Endodontics, UR Anoop Research Group, Puducherry, IND; 7 Periodontics, UR Anoop Research Group, Puducherry, IND; 8 Dentistry, UR Anoop Research Group, Puducherry, IND

**Keywords:** parkinson' s disease, novel drug delivery, drug delivery system, maxillofacial route, levodopa

## Abstract

Parkinson's disease is characterized by the loss of nigrostriatal dopaminergic neurons in the brain. Dopamine cannot be administered systemically because it does not cross the blood-brain barrier. Oral levodopa remains the gold standard to date. Currently, for patients who show a poor response to oral levodopa and for those who cannot take it orally, the alternate routes available are inhalation and continuous administration via intestinal and subcutaneous routes. In this report, a novel maxillofacial route was used for the first time in the world to administer levodopa to a Parkinson's patient. Furthermore, the efficacy of maxillofacial administration was compared with the oral route of administration.

## Introduction

Parkinson's disease is the second most common neurodegenerative disorder, affecting more than 1% of the population above 65 years of age. The prevalence is expected to double by 2030 [1]. According to the WHO factsheet (dated August 9, 2023) on Parkinson's disease, the incidence increases with age and affects males more often than females. The prevalence of Parkinson's disease has doubled in the past 25 years. Global estimates in 2019 showed over 8.5 million individuals with Parkinson's disease.

Oral levodopa remains the gold standard in the treatment of Parkinson's disease [2,3,4,5]. Levodopa is decarboxylated in the gut, and about 30% of it crosses the intestinal mucosa. The absorbed levodopa is further metabolized by the liver, kidneys, and brain capillary endothelium. The effectiveness of levodopa depends on its metabolism to dopamine in the brain. However, only about 1% of oral levodopa crosses the blood-brain barrier [6].

Levodopa provides good symptom relief in the initial years. As the disease progresses, the effect of levodopa diminishes, and motor complications occur. Such patients require more levodopa at shorter intervals. As the therapeutic window narrows with disease progression, higher doses result in levodopa-induced dyskinesia [7]. High levels of levodopa metabolites cause orthostatic hypotension, nausea, and vomiting [6].

For patients who show a poor response to oral levodopa or cannot take it orally, alternate routes like oral inhalation, intestinal, and subcutaneous are available. However, these routes have limitations. The adverse events associated with the intestinal route include device-related surgical complications, infection, blockage of the tube, pancreatitis, bleeding into the intestines, and sleep attacks. Subcutaneous administration has been linked to nodules and pain. Adverse events associated with oral inhalation include cough and upper respiratory tract infection [2,3].

Therefore, a simple technique that can deliver low doses of levodopa by bypassing the gut, first-pass metabolism in the liver, and the blood-brain barrier would be useful for patients. In this report, we present a novel maxillofacial technique and compare it with the oral route of levodopa administration.

## Case presentation

An elderly male patient in his late 80s with idiopathic Parkinson's disease presented at the Movement Disorders Clinic at the Department of Neurology, Indira Gandhi Government General Hospital and Post Graduate Institute, Puducherry, India.

On clinical examination, the vitals were stable; blood pressure was 120/80 mm Hg with no postural fall, and pulse was 70/minute, regular in volume and rhythm. Higher mental function was normal with an MMSE score of 28/30. Cranial nerve examination and spino-motor system examination were normal. Bulk and power were 5/5 for all four limbs. The patient exhibited cogwheel rigidity in both upper and lower limbs, more distally. Deep tendon reflexes were normally elicitable, and plantar response was flexor bilaterally. Extrapyramidal system examination showed mask-like facies with decreased blink rate. Rest and postural tremors were more pronounced on the left side compared to the right, and the patient also had a short-stepping gait with festination, en bloc turning, and a positive pull test. The patient met the UK Brain Bank criteria for Parkinson's disease and was initially started on Syndopa at 110 mg twice a day, increased to thrice a day after one year. Neuroimaging, including a CT scan of the brain, was normal, and the patient responded well to Syndopa.

**Figure 1:**
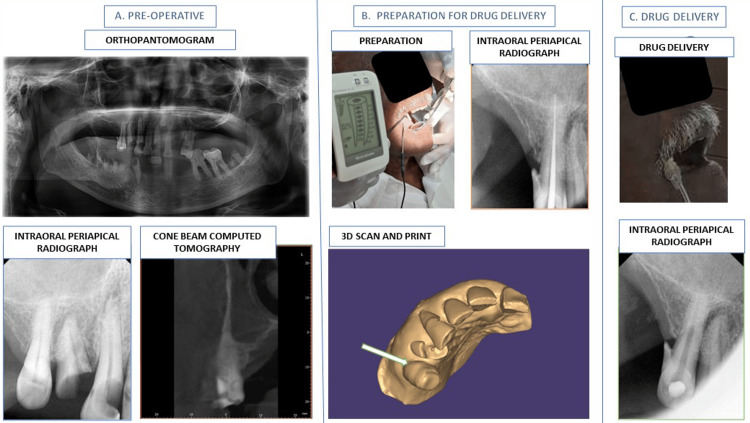
Maxillofacial drug delivery Administration of levodopa through the maxillofacial route. (A) Two-dimensional (orthopantomogram and intraoral periapical) and three-dimensional (cone beam computed tomography) radiographic views of the upper-right second premolar. The root tip of the tooth is in close proximity to the floor of the maxillary sinus. (B) The preparation of the root canal of the upper right second premolar till the tip of the root. The digital scan of the tooth is used for 3D printing of the drug delivery system. (C) Maxillofacial drug delivery. The post-operative intraoral periapical X-ray of the upper right second premolar is normal.

The upper right second premolar was non-vital. Radiographic examination revealed that the apex of the non-vital tooth was in close proximity to the floor of the maxillary sinus. With the patient's consent, root canal treatment was initiated; the pulp cavity was debrided, cleaned, and enlarged. A digital scan of the treated tooth was made. A 3D-printed drug delivery system that could be attached to the tooth was designed to administer the drug through the tooth (Figure 1A-1C).

A washout period of 7.5 hours was maintained between the last dose of oral levodopa/carbidopa and drug administration at the hospital. On Day 1, the patient had breakfast one hour after the oral dose of levodopa/carbidopa tablet. On Day 2, the patient had breakfast after receiving 1/20th of the oral dose of levodopa through the maxillofacial route. The drug delivery system was removed after drug administration. The breakfast menu was the same on both days.

Blood samples were collected, and UPDRS (Unified Parkinson's Disease Rating Scale, Part 3, the motor component) scores were assessed at pre-dose and at intervals of 30, 60, 90, and 120 minutes post-dose. The blood samples were centrifuged, and the plasma was stored at -80°C.

**Figure 2:**
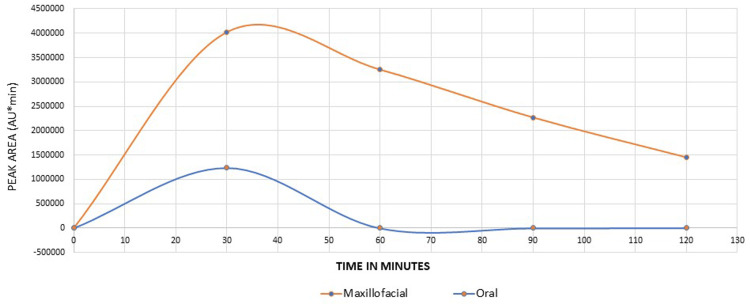
HPLC-plasma values of levodopa Comparison of plasma values of levodopa-maxillofacial administration of levodopa versus oral administration of a tablet of levodopa/carbidopa. The Y-axis shows the HPLC peak area in AU*min (absorbance units × minutes). The X-axis shows time in minutes after administration of levodopa. HPLC: high-performance liquid chromatography.

The blood samples were analyzed using HPLC (high-performance liquid chromatography). Blood levels of levodopa after oral tablet administration peaked at 30 minutes and declined to negligible levels by 60 minutes. In contrast, the maxillofacial route showed a higher peak at 35 minutes, with levels decreasing from 60 to 120 minutes (Figure 2).

**Figure 3:**
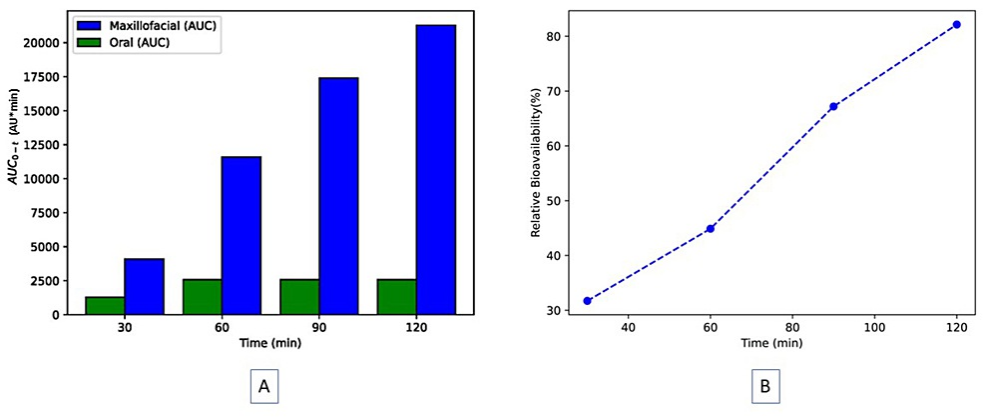
Relative bioavailability of levodopa Maxillofacial administration of levodopa versus oral administration of a tablet of levodopa/carbidopa. (A) Comparison of the HPLC area under the curve (AUC) of oral tablet of levodopa/carbidopa with maxillofacial administration of levodopa. The Y-axis shows the HPLC peak area in absorbance units × minutes. The X-axis shows time in minutes after administration of levodopa. (B) The relative bioavailability of levodopa following maxillofacial administration with respect to time.

The pharmacokinetic analysis was conducted using the linear trapezoidal method, and the area under the concentration-time curve was calculated. The relative bioavailability of the maxillofacial route was consistently higher in the plasma, starting at the 5th minute and maintained through the 120th minute (Figure 3).

**Figure 4:**
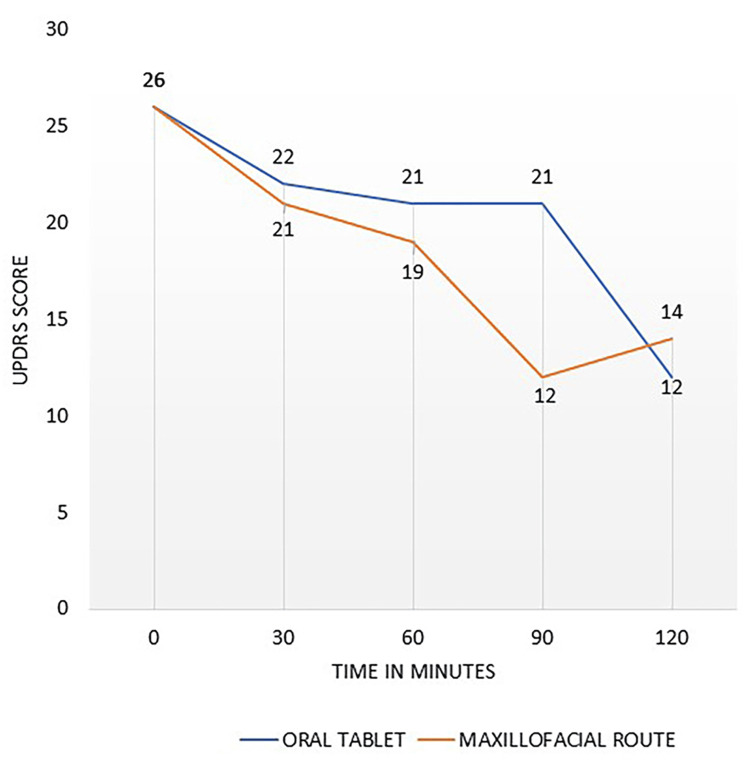
UPDRS scale Maxillofacial delivery of levodopa versus oral tablet of levodopa/carbidopa. UPDRS: Unified Parkinson's Disease Rating Scale.

The motor component of UPDRS showed a one-point difference at the 30th minute favoring the maxillofacial route. At the 60th minute, it improved by two more points. When compared with the oral route, the best scores in favor of the maxillofacial route were noted at the 90th minute. A relatively high plasma value at 30th minute following maxillofacial administration correlated only with mild clinical improvement in UPDRS, motor component. But subsequently the patient had good clinical improvement till 90th minute as noted in UPDRS scores, which also corresponded with the high plasma values following maxillofacial administration (Figure 4). The UPDRS score was higher for the oral route and lower for the maxillofacial route.

This result may suggest that the maxillofacial route can be useful for frequent low dosing to prevent the "off" phenomenon as well as dyskinesia. The efficacy of the maxillofacial route as an on-demand treatment for early morning "off," delayed "on," and "no on" requires further evaluation and assessment, because the clinical improvement at the 30th minute was not high in the maxillofacial route when compared with the oral route, despite having high plasma values. As this study is limited to one patient, a clearer understanding will emerge when frequent doses of levodopa are administered to more patients to measure the exact outcome.

## Discussion

Following maxillofacial administration of levodopa, the plasma value of levodopa was higher and peaked at 35 minutes, compared with the oral route. It then gradually decreased from 60 minutes to 120 minutes. The relative bioavailability of levodopa in the plasma following maxillofacial administration was consistently higher from the 5th minute to the 120th minute. When compared with oral administration of levodopa, the UPDRS motor scores showed better motor scores following maxillofacial administration and also correlated with higher levodopa plasma values. The maxillofacial technique provides a novel route for administering levodopa. If these results are extrapolated to more patients, the new route could be useful for patients who cannot take levodopa orally and in those who show poor response to oral levodopa.

Oral levodopa is the gold standard in the treatment of Parkinson's disease [4]. It is absorbed in the proximal one-third of the small intestine. Between 30% and 100% of Parkinson's patients suffer from impaired gastric motility. Delayed delivery of levodopa to the intestinal absorption sites results in increased pre-systemic peripheral decarboxylation, reduced levodopa absorption, delayed "on," dose failure, medication overload, and motor complications [5].

Levodopa has a short half-life of about 50 minutes, which is increased to approximately 90 minutes by the addition of peripheral enzyme inhibitors [6]. Levodopa is effective in the early stages of the disease, likely due to the preserved capacity of the presynaptic nerve terminals to store dopamine. The effect of levodopa progressively diminishes with the continued loss of neurons in the substantia nigra. This can initially be improved by increasing the frequency of levodopa doses. With further progression, motor fluctuations, "on/off" phenomena, and dyskinesias appear, which may not respond to increased frequency of levodopa doses. The fluctuating plasma levels of levodopa result in akinesia at subtherapeutic levels and dyskinesias at peak levels. With further progression, dyskinesias become more complex, with practically no response to levodopa [7].

Clinical evidence suggests that continuous delivery of levodopa can reduce motor complications. Continuous oral delivery of levodopa/carbidopa showed less plasma variability and reduced "off" time compared with standard intermittent oral levodopa/carbidopa therapy [8]. Controlled-release oral formulations exhibit sustained plasma levels compared to standard levodopa/carbidopa. The drawbacks include lower bioavailability, longer time to peak, delayed onset of clinical response, and higher daily dose despite a lower frequency of dosing [2,3].

The intraduodenal route bypasses the stomach and delivers levodopa directly to its absorption sites in the small intestine, resulting in faster and more stable plasma concentrations when compared with oral dosing. However, surgical complications are a disadvantage with this route. Recent studies have identified continuous subcutaneous levodopa/carbidopa as a feasible route [2,3], although this route is not yet approved.

Recently, an inhalable formulation of levodopa has been approved for intermittent treatment of "off" periods in patients treated with levodopa/carbidopa. Oral inhalation bypasses the gut, and levodopa is delivered into the lungs for rapid absorption into the systemic circulation. It is not recommended for patients with asthma or other chronic lung diseases. Cough was the most common adverse event. Improvement in UPDRS-III score was noted at 10 minutes and was sustained until 60 minutes [9]. Orally inhaled levodopa without a dopa decarboxylase inhibitor was compared with oral levodopa/carbidopa. Inhaled levodopa was absorbed faster than oral levodopa, with a bioavailability of 53% [10]. The faster absorption helped in bridging the "off" periods, as demonstrated in two studies [11,12].

Patients will ultimately require device-assisted therapies to manage motor fluctuations [13]. Recently, in vivo animal studies have shown that drugs can be successfully delivered into the brain through the maxillofacial route via the paravascular, vascular, perineural, lymphatic, and glymphatic pathways [14,15,16].

The patient's Hoehn and Yahr stage at the time of presentation was 3. The frequency of untreated caries in Parkinson's patients is reported to be high at Hoehn and Yahr stage II and above [17]. Patients with caries involving the pulp will require root canal treatment. The maxillofacial route was selected because higher bioavailability could be achieved at lower doses by bypassing the gut and first-pass metabolism. The limitations of the maxillofacial route include the requirement for a locally invasive procedure like a root canal in the upper posterior teeth, as well as potential for infection and pain. In this case, the patient had a non-vital upper posterior tooth that required a root canal. He tolerated the procedure well without any adverse events.

Therefore, the maxillofacial technique can be an alternative to the oral route in Parkinson's patients (Table 1).

**Table 1:** Comparison of routes for levodopa drug delivery

Oral route	Oral inhalation route	Maxillofacial route
Decarboxylated in the gut. Absorption affected by impaired gastric emptying.	Avoids metabolism in the gut. Absorption not affected by impaired gastric emptying.	Avoids metabolism in the gut. Absorption not affected by impaired gastric emptying.
Larger doses required.	Low doses required.	Low doses required.
Non-invasive.	Non-invasive.	Patient preparation required prior to drug delivery.
Slow absorption, slow onset of action.	Faster absorption, Faster onset of action.	Faster absorption, Faster onset of action.
Drug delivery cannot be controlled.	Drug delivery cannot be controlled.	Drug delivery can be controlled.
Not affected by chronic respiratory conditions. Affected by dysphagia, conditions involving the stomach and the proximal intestine.	Affected by chronic respiratory conditions. Not affected by dysphagia, conditions involving the stomach and the proximal intestine.	Not affected by chronic respiratory conditions. Not affected by dysphagia, conditions involving the stomach and the proximal intestine.

## Conclusions

This novel route can be useful for patients with dementia, bedridden patients who require assistance for daily activities, patients in the ICU with a Ryle's tube in situ, and patients with dyskinesias where frequent low doses will be required to attain a steady state in the plasma. It can also serve as an on-demand treatment option for early morning "offs," end-of-dose wearing "offs," delayed "on," suboptimal "on," and dose failure. Further studies are required.
